# Impact of Interprofessional Student Teams at a Remote Area Medical Event in Rural Appalachia

**DOI:** 10.13023/jah.0502.06

**Published:** 2023-08-01

**Authors:** Emily K. Flores, KariLynn Dowling, Caroline Abercrombie, Rick L. Wallace

**Affiliations:** East Tennessee State University, florese@etsu.edu; East Tennessee State University; East Tennessee State University; East Tennessee State University

**Keywords:** Appalachia, attitudes, health professions education, interprofessional education, mixed methods, Remote Area Medical, rural health

## Abstract

**Introduction:**

Education in interprofessional collaboration is vital to expand healthcare access, especially in areas of higher disparity. To address this need, interprofessional faculty collaborators incorporated undergraduate and graduate health profession students into teams at an annual Remote Area Medical event in rural Appalachia between 2017 and 2020.

**Purpose:**

This article evaluates the impact of an interprofessional student teams model on both patient care experience and students’ interprofessional collaboration attitudes and behaviors.

**Methods:**

Student volunteers completed pre- and post-event surveys containing questions about demographics, open-ended questions, and questions from two instruments: the Student Perceptions of Interprofessional Clinical Education-Revised Instrument, Version 2 (SPICE-R2) and the Interprofessional Collaborative Competency Attainment Scale-Revised (ICCAS-R). Quantitative data were analyzed statistically; qualitative data thematically. Tally forms collected patient care interventions that were compared to regional health disparities. Two years of survey data and four years of intervention data were analyzed.

**Results:**

There was an increase (*p* < 0.001) in the post-event survey SPICE-R2 factors (teamwork, healthcare outcomes, and roles and responsibilities) in 2020 but not in 2019. ICCAS-R mean post-event composite scores increased (*p* < 0.05) in both 2019 and 2020. Qualitative coding of open-ended responses revealed interprofessional competency themes and provided event feedback. Over 5,900 health-disparity-focused interventions were completed between 2017 and 2020.

**Implications:**

Students participating in interprofessional teams demonstrate changes in attitudes towards the interprofessional approach to care, an improved ability to collaborate interprofessionally, and a positive impact on patient care interventions. The findings allow educators to understand how experiential interprofessional education influences students’ interprofessional attitudes and beliefs while benefitting patient care.

## INTRODUCTION

The Appalachian Region ranks worse than the rest of the nation in mortality related to heart disease, cancer, COPD, injury, stroke, diabetes, and diseases of despair.^1^,^2^ Elevated years of potential life lost and negative social determinants of health have persisted over time.^1^,^2^ Tennessee, the site of this report, ranked 42^nd^ in the country in health factors in 2018.^3^ There is significant need for advancement of healthcare services in this region.

Interprofessional care is gaining emphasis as a method to expand healthcare access, especially in rural and underserved populations where disparities and health professional shortages are greatest.^1^ As interprofessional practices grow within health systems, current health profession students see interprofessional collaboration as a means of overcoming barriers in rural areas.^4^ Educators must provide opportunities for exposure to and application of interprofessional principles in health professions education.

Formal interprofessional education (IPE) occurs when students from two or more professions learn about, from, and with each other to enable effective collaboration and improve health outcomes in an educational setting.^5^ The four core competencies of IPE are (1) *Values/Ethics for Interprofessional Practice*, (2) *Roles/Responsibilities*, (3) *Interprofessional Communication*, and (4) *Teams and Teamwork*.^5^ Ideally, health professional students take a baseline achievement of these core competencies with them upon graduation, promoting dissemination and sustainment of interprofessional practice.

Engaging with interprofessional teams in an experiential environment is one way to support students in professional identity formation while allowing them to better understand other professions, identify roles in patient care, and improve readiness for interprofessional collaboration.^6^–^9^ In caring for rural and underserved communities, graduate-level, community-based IPE models have been utilized in this manner,^10^–^12^ and positive patient outcomes have been observed.^13^ Rural programs may also expand student awareness of rural community culture and needs.^14^

East Tennessee State University (ETSU)’s regional healthcare, education, and research endeavors are collectively referred to as ETSU Health, and there is a specific emphasis on primary care and rural healthcare delivery.^15^ Formal IPE programs have grown under leadership of the ETSU Center for Interprofessional Education and Collaboration. Mindful of the benefits of IPE in experiential environments, ETSU Health faculty partnered with Remote Area Medical (RAM) to implement a new model utilizing interprofessional teams of graduate and undergraduate student volunteers at a RAM event in Gray, Tennessee, beginning in 2017.

RAM is a nonprofit provider of free mobile clinics that provide underserved and uninsured patients with dental and vision care and address their chronic medical conditions, utilizing volunteer professionals and general support.^16^ Patients generally come to an event seeking dental or vision care, but due to Appalachian regional health disparities, often need additional services. Local RAM partnerships connect patients to ongoing educational and healthcare resources within their community. RAM works with communities allowing a blend of local distinctiveness within its organizational structure, and it is a prime opportunity for interprofessional education and practice.

This applied research describes a model for incorporating health profession students into teams at a RAM event in Northeast Tennessee and examines the impact of participation on both student attitudes and patient care. The number of health professions represented, and the inclusion of undergraduate students, are factors unique to this intervention. Additionally, the utilization of mobile teams is a novel approach to partnership with RAM, though past partnership activities with RAM have been positive.^10^,^17^–^19^

## METHODS

A cross-sectional, longitudinal study format with mixed methods including quantitative and qualitative components was utilized. This research was reviewed by the ETSU Institutional Review Board.

### Participants

Students enrolled in undergraduate and graduate health profession programs at the academic partner were invited to volunteer as a part of interprofessional teams at the RAM event. Each team represented at least three different professions, included a variety of student levels, a variety of professions, and was precepted by a pair of interprofessional preceptors from participating programs.

### Setting

The RAM event was held at a fairground facility consisting of multiple service areas. Interprofessional teams served throughout the event facility, partnering with each service area to fill patient care needs and offer expanded care and education. Interprofessional activities, such as a team huddle, handoffs, and debriefing, were incorporated into team activities.

### Data Collection

A voluntary, electronic survey including consent for participation in research was administered to student volunteers via REDCap prior to and following the 2019 and 2020 RAM events. Students could complete the pre-event survey at home via a link on the pre-event training website (up to three weeks prior) or by utilizing a provided device as they arrived at the event. Students could complete the post-event survey via a provided device at the end of their last shift or via an email invitation following the event (up to one week after). A participantgenerated unique identifier was utilized to link pre- and post-event surveys allowing for anonymous responses.

The pre-event survey was estimated to take less than five minutes to complete and consisted of demographic questions and the *Student Perceptions of Interprofessional Clinical Education-Revised Instrument, Version 2* (SPICE-R2).^20^ The post-event survey was estimated to take approximately 10 minutes to complete, as it also included the *Interprofessional Collaborative Competency Attainment Scale-Revised* (ICCAS-R) and open-ended questions.^21^ The evaluation instruments were selected from previously validated IPE surveys to provide a comprehensive picture of students’ self-evaluated interprofessional attitudes and skills before and after event participation. SPICE-R2 is comprised of 10 items on a five-point Likert scale (strongly disagree to strongly agree) evaluating students’ perceptions across three IPE factors: interprofessional teamwork, roles and responsibilities for collaborative practice, and patient outcomes from collaborative practice. ICCAS-R contains 20 items on a five-point Likert scale (poor to excellent) that evaluate students’ self-perceived ability to perform tangible interprofessional team skills in a retrospective, pre-test/post-test fashion, where students answer each question twice from a before-event and after-event perspective. The post-event survey’s open-ended questions were developed based on the event experience, application to future practice, and areas for improvement.

Interprofessional student teams tracked patient interventions utilizing a paperbased *Intervention Log*. Two years of survey data and four years of intervention data were analyzed.

### Data Analysis

Statistical Package for the Social Sciences (SPSS), version 25, was utilized for quantitative analysis. Descriptive statistics were calculated for all demographics. Per instructions for the validated survey instruments, composite scores were calculated for each of the three SPICE-R2 factors using mean responses to corresponding items, and an overall composite score was calculated for the ICCAS-R from mean responses to all 20 items.^20^,^21^ Analysis of changes in the SPICE-R2 factor composite scores and in ICCAS-R composite scores from preevent evaluation to post-event evaluation was conducted using paired sample ttests. Independent sample t-tests were utilized to evaluate SPICE-R2 and ICCASR scores for underlying differences between demographic subgroups. For all ttests, the significance level was set at an *α* of 0.05.

Qualitative responses from the surveys were analyzed by multiple coders for emerging themes.^22^ Open-ended (OE) question themes were developed from inductive codes that had frequencies greater than five. Codes with frequencies of 10 or more were considered major themes.

Data was tabulated from the *Intervention Log* and categorized to describe the impact of student volunteers on quantity and type of patient care interventions offered at each annual event, 2017–2020. Intervention types were then compared to regional health disparities.

## RESULTS

### Participant Demographics

**Figure 1:**
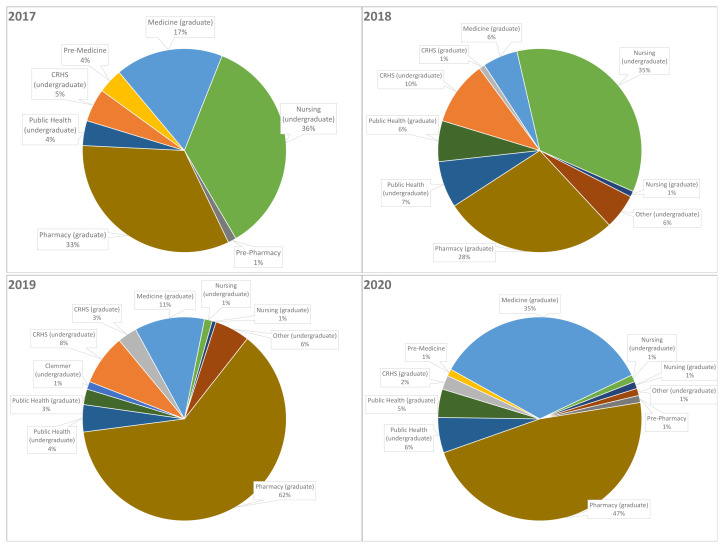
Interprofessional Student Distribution NOTE: 2017 student volunteers N = 76 (87 student experiences); 2018 student volunteers N = 108 (124 student experiences); 2019 student volunteers N = 162 (183 student experiences); 2020 student volunteers N = 89 (93 student experiences); CRHS = College of Rehabilitative Health Sciences; Clemmer College = Counseling, Education, Leadership, and Sports degree programs

The interprofessional team distribution varied each year (Fig. 1). Of the seven types of students participating in 2017, undergraduate nursing and graduate pharmacy students each made up approximately one-third. These groups remained the largest student representation in 2018 out of nine types of students. In 2019 and 2020, of the 10 student types, graduate pharmacy students were a significant majority, with graduate medical students joining this majority in 2020. Students were allowed to participate on more than one day of the event; therefore, there is a higher number of student experiences than student volunteers. Each year, about half of on-site preceptors were pharmacist faculty, complemented by preceptors from Counseling, Medicine, Medical Library, Nursing, Nutrition, Occupational Therapy, Physical Therapy, Public Health, Respiratory Therapy, Social Work, and Speech-Language Pathology programs. The event in 2017 had 11 preceptors; 2018 had 23; 2019 had 22; and 2020 had 22, despite COVID-19. Hours volunteered began to be logged in 2018, showing a contribution of 426.32 student hours and 195.87 preceptor hours, resulting in a ratio of 2.18 students to preceptor per hour. In 2019, it was 713.07 student hours and 201.35 preceptor hours, with a ratio of 3.54. In 2020 it was 460.18 student hours and 175.15 preceptor hours, with a ratio of 2.63.

### Quantitative Survey Findings

Out of 162 student volunteers in 2019, 107 pre-event survey responses and 108 post-event survey responses were obtained (response rate = 66%). Out of 89 student volunteers in 2020, 79 pre-event survey responses and 79 post-event survey responses were obtained (response rate = 94%). For both 2019 and 2020, the majority of survey respondents were female and between ages 21 and 25 years (Table 1). Approximately one-quarter of the student volunteers were originally from a rural area (26.9% in 2019; 22.8% in 2020) and nearly one-third had an immediate family member in another health profession (32.4% in 2019, 30.4% in 2020). Graduate students made up 75.7% of the cohort in 2019 and 88% of the cohort in 2020, with approximately one in two in the first half of their graduate program. Compared to 2019 respondents, more students in 2020 had previous IPE experiences, had graduated from the ETSU IPE program, and had participated in RAM interprofessional student teams in previous years.

Linking participant-generated, unique identifiers from pre- and post-event survey responses resulted in 70 valid matched responses from 2019 and 69 valid matched responses from 2020 for analysis of the SPICE-R2. In 2019, numerical increases were observed in the mean SPICE-R2 factor scores from pre-event survey to post-event survey; however, there were no statistically significant changes in the factors among the overall respondents (Table 2), and only two subgroups demonstrated statistically significant improvements in any of the SPICE-R2 factors. Students who did not report having an immediate family member in another health profession (N = 51) increased their mean score on the interprofessional teamwork factor from 4.43 (SD = 0.78) to 4.59 (SD = 0.68; *p* = 0.029), and students who were not currently enrolled in the formal ETSU IPE program (N = 25) increased their mean score on the roles and responsibilities factor from 4.0 (SD = 0.65) to 4.15 (SD = 0.57; *p* = 0.046) in 2019. In 2020, statistically significant increases in all mean SPICE-R2 factor scores (*p* < 0.001) were observed among respondents.

There were 104 complete post-event survey responses analyzed for the ICCAS-R in 2019 and 79 in 2020. Statistically significant increases in ICCAS-R composite scores were observed after the RAM event both years (see Table 2), and these increases were consistent across demographic subgroups.

### Qualitative Survey Findings

Codes with frequencies of 10 or more were considered major themes for the question (Table 2). The years 2019 and 2020 displayed overlap in qualitative themes, with students acknowledging realization of *Patient/Community Need* (OE1, OE2, OE6), available *Resources/Services* (OE1), and growth in *Specific Professional Skills* (OE2), such as learning how to check a blood glucose or take a medication history from an interprofessional student colleague. Themes revealed advancement in the interprofessional competencies of *Roles/Responsibilities* (OE2, OE3, OE4) and *Teams/Teamwork* (OE2, OE3, OE4), as well as growth in *Interprofessional Perspective/Respect* (OE3, OE4). For OE5 the highest coded major theme was *No Challenge/Obstacles* while working in the teams; however, additional themes included *Roles/Responsibilities*, *Interprofessional Communication*, and *Event Organization/Flow* with specific acknowledgments of COVID-19 adjustments on the event in 2020.

### Interventions

Each year prior to 2020, this event served over 750 patients, provided over 1,000 encounters, and offered services valuing over $485,000 (Table 3). All were reduced in 2020 due to COVID-19. The number of interventions per patient decreased from 2017 (2.60) to 2018 (1.66) but increased in 2019 (1.94) and 2020 (5.07). With the addition of logging hours of service in 2018, there were 2.65 interventions per student hour in 2018, 2.12 interventions per student hour in 2019, and 2.63 interventions per student hour in 2020. Medication histories and health screens consistently yielded high intervention numbers each year. COVID-19 screening was a new skill introduced in 2020 and yielded high intervention numbers. Across the years, the most frequent patient education provided was on immunization, smoking cessation, and anxiety/depression. Blood glucose, diabetes, and diet/exercise education were also provided in high numbers most years. Discharge medication education in the dental area was introduced in 2020, which correlated with an increase in dental health education, as well. Additionally, Medical Library staff coordinated with the teams and other RAM patient care services to conduct numerous, individualized literature searches each year and provide patient educational handouts or packets. Over time, advance preparation has been refined to have deliverables available that correlate with common patient needs and the services the teams and medical library staff commonly discuss with patients.

## DISCUSSION

Lazar et al. have described the complexities of treating patients at other RAM events in the Appalachian Region similar to what students and providers find each year at this event.^23^ Reviews on teaching interprofessional teamwork skills show that most activities where interprofessional teams interact result in positive changes in student perceptions and attitudes towards IPE and practice.^9^,^24^,^25^ The study demonstrates that a well-planned IPE approach at a RAM event by an academic partner was mutually beneficial to the RAM event and interprofessional students by allowing demonstration of one or more interprofessional competencies, positively impacting student attitudes toward interprofessional practice, and increasing the number and types of clinical interventions at the event.

This IPE experience was intentionally developed in partnership with RAM and implemented with significant planning and collaboration. The teams were organized and placed strategically to identify and meet needs of the patients present. Students provided over 1.5 interventions per patient each year, allowing patients to take advantage of more available services and resources. The provided interventions—including referrals for naloxone rescue training, immunizations, and education—aligned with known regional health disparities.^1^,^2^ Authors attribute the decrease in interventions per patient from 2017 to 2018 to an increase in services provided by other organizations, particularly blood glucose measurements and education, offsetting the need for services done exclusively by our teams in the event’s first offering. The intervention per patient increase in 2019 is attributed to teams completing more health screens and, in turn, completing more referrals to available services. Interventions per patient increased in 2020 with teams taking on COVID-19 screening, which increased student–patient interaction while providing a needed service.

Students were able to learn with and from each other about the needs and resources in the region, as demonstrated by the *Patient/Community Need* and *Resources/Services* themes identified in open-ended questions. These included responses about available services/resources both at the RAM event and within the community. Assistance from preceptors and on-site translators helped address student-reported challenges with *Patient Communication/Literacy* related to non-English speakers and gaps in health literacy. Demonstration of interprofessional competencies was evident in that emergent inductive codes of student responses matched published interprofessional competencies.

In evaluating the impact on student attitudes, the significant difference in SPICE-R2 factors in 2020 that was not found in 2019 could be attributed to the addition of intentional preceptor training, tools, and guidance that were incorporated in the 2020 event. The significant improvement seen in interprofessional collaborative behaviors as measured by the ICCAS-R could be attributed to students having the opportunity to utilize interprofessional skills in a real-world setting beyond their classroom training. A positive impact of this specific activity on improving student self-assessment of interprofessional collaborative behaviors was demonstrated. Of note, though being educated in an interprofessional environment at ETSU Health, most participants did not have past experience with the RAM interprofessional teams, which may decrease confounding of survey results from past similar experiences.

Limitations include loss of data due to unmatched survey responses, which may be reduced with simplification of the participant-generated identifier in future iterations; the potential for response shift bias with SPICE-R2; the potential for recall bias with the ICCAS; and student self-reported event evaluation in openended questions. Authors also cannot account for differences that may be attributed to changes in the interprofessional education curriculum due to COVID-19 and ongoing quality improvement.

## IMPLICATIONS

These results show that student confidence in their ability to perform in an interprofessional team increased after participating and that they made an appreciable impact towards the efforts of the RAM clinic each year. These findings suggest that interprofessional student experiences like this promote interprofessional competency while improving students’ self-reported readiness and familiarity with interprofessional collaboration. Ultimately, students reported that the RAM event provided a good experience for them to understand the impact they can have as healthcare providers while serving their rural community. A successful event requires significant preparation and ongoing quality improvement. This model provides a framework for community engagement opportunities that integrate impactful IPE clinical experiences into an existing community event to increase patient interventions. Educators are encouraged to prepare future health professionals for interprofessional practice by pairing experiential education with increased healthcare service and access in communities of need.

**SUMMARY BOX**

**What is already known about this topic?**

Interprofessional practice models are increasingly being implemented in healthcare settings, particularly for rural and underserved populations, to address gaps in healthcare access. There is a corresponding need to provide interprofessional practice exposure and education to future health professionals; various interprofessional service and learning activities reported in the literature have resulted in positive changes in student perceptions and attitudes towards interprofessional practice.

**What is added by this report?**

This report details and evaluates a novel, mobile student/faculty interprofessional teams model that partners with an annual Remote Area Medical (RAM) free clinic event. This model has incorporated up to ten types of health professions and includes undergraduates. Students demonstrated improvements in self-reported positive perceptions and competency for interprofessional collaboration, in addition to meaningful contributions to health care for rural community members complementary and additive to the baseline services provided by RAM.

**What are the implications for future research?**

Future research designs may focus on long-term impacts of interprofessional service during healthcare professional education as well as cost–benefit of student service for the partnering service organization.

## Supplementary Information



## Figures and Tables

**Table 1:** 2019 Student Demographics (N = 108) and 2020 Student Demographics (N=79)

Demographic category	2019	2020
**Gender**		
Male	33 (30.6%)	19 (24.1%)
Female	75 (69.4%)	59 (74.7%)
Other	–	1 (1.3%)
**Age group (years)**		
18 to 20	19 (17.6%)	4 (5.1%)
21 to 25	67 (62%)	54 (68.4%)
26 to 30	13 (12%)	11 (13.9%)
31 to 35	6 (5.6%)	6 (7.6%)
Over 35	3 (2.8%)	4 (5.1%)
**Hometown size**		
Rural	29 (26.9%)	18 (22.8%)
Small Town	49 (45.4%)	33 (41.8%)
Suburb	21 (19.4%)	21 (26.6%)
Large City	9 (8.3%)	7 (8.9%)
**Profession of study**		
Allied Health	3 (2.8%)	1 (1.3%)
Audiology	1 (0.9%)	0
Medicine	12 (11.1%)	28 (35.4%)
Nursing	(3.7%)	3 (3.8%)
Pharmacy	67 (62%)	37 (46.8%)
Physical Therapy	5 (4.6%)	0
Public Health	7 (6.5%)	6 (7.6%)
Speech Pathology	2 (1.9%)	1 (1.3%)
Other	7 (6.5%)	3 (3.8%)
**Progress towards current degree** *		
Undergraduate first half of degree	8 (7.5%)	2 (2.5%)
Undergraduate last half of degree	18 (16.8%)	7 (8.9%)
Graduate first half of degree	57 (53.3%)	39 (49.4%)
Graduate last half of degree	24 (22.4%)	31 (39.2%)
**Immediate family member in a different health profession?**		
Yes	35 (32.4%)	24 (30.4%)
No	73 (67.6%)	55 (69.6%)
**No. past IPE experiences**		
None	44 (40.7%)	17 (21.5%)
1 to 3	53 (49.1%)	31 (39.2%)
4 to 6	5 (4.6%)	12 (15.2%)
7 to 9	0	8 (10.1%)
10 or more	6 (5.6%)	11 (13.9%)
**Currently enrolled in ETSU IPE program?**		
Yes	71 (65.7%)	43 (54.4%)
No	37 (34.3%)	36 (45.6%)
**Graduated from ETSU IPE program?**		
Yes	8 (7.4%)	25 (31.6%)
No	100 (92.6%)	54 (68.4%)
**Past participation in RAM Interprofessional Teams?**		
Yes	16 (14.8%)	26 (32.9%)
No	92 (85.2%)	53 (67.1%)

NOTES: IPE = Interprofessional education

* 2019: 107 responses; 2020: 79 responses

**Table 2:** Survey Results, 2019 and 2020

Quantitative Findings						
	2019 Pre-event survey M (SD)	2019 Post-event survey	2019 *p*-value	2020 Pre-event survey	2020 Post-event	2020 *p-*value
**SPICE-R2**	N=70			N=69		
Interprofessional Teamwork Factor	4.50 (0.72)	4.54 (0.72)	0.568	4.67 (0.43)	4.74 (0.41)	<0.001*
Roles and Responsibilities for Collaborative Practice Factor	4.00 (0.81)	4.09 (0.81)	0.374	4.13 (0.73)	4.49 (0.52)	<0.001*
Patient Outcomes from Collaborative Practice Factor	4.36 (0.76)	4.40 (0.77)	0.650	4.49 (0.55)	4.68 (0.50)	<0.001*
**ICCAS-R**	N=104			N=79		
Overall Composite Score	3.65 (0.80)	4.03 (0.73)	<0.001*	4.19 (0.65)	4.59 (0.50)	<0.05*
**Qualitative Themes**						
**November 2019**			**November 2019**			
**OE1: ** ***What surprised you while volunteering at the Gray, Tennessee RAM event?***						
Patient/Community Need† (22)			Patient/Community Need† (17)			
Resources/Services† (13)			Resources/Services† (16)			
Volunteer Volume/Willingness† (12)			Patient Volume (-) (8)			
Impact (5)			Event Organization/Flow (7)			
Kindness (5)			Teams/Teamwork (5)			
Event Organization/Flow (5)			Volunteer Volume/Willingness (5)			
Patient Engagement (5)			COVID-19 Pandemic (5)			
Patient Variety/Diversity (5)						
**OE2: ** ***What is something that you learned from participating in interprofessional student teams at the Gray, Tennessee RAM event?***						
Specific Professional Skills† (20)			Roles/Responsibilities† (15)			
Teams/Teamwork† (14)			Teams/Teamwork† (15)			
Patient/Community Need† (10)			Specific Professional Skills† (13)			
Roles/Responsibilities (6)			Impact (7)			
Volunteer Volume/Willingness (6)			Patient/Community Need (7)			
Interprofessional Communication (5)						
Resources/Services (5)						
**OE3: ** ***How did working in an interprofessional student team change your idea of the roles of other health professionals?***						
Roles/Responsibilities† (22)			Roles/Responsibilities† (22)			
Interprofessional Perspectives/Respect† (17)			Interprofessional Perspectives/Respect† (15)			
Teams/Teamwork† (12)			Teams/Teamwork† (13)			
**OE4: ** ***How did working in an interprofessional student team impact you as an individual within your chosen profession?***						
Professional Satisfaction† (20)			Professional Satisfaction† (15)			
Roles/Responsibilities† (13)			Interprofessional Perspectives/Respect† (11)			
Teams/Teamwork (7)			Teams/Teamwork† (10)			
Interprofessional Communication (6)			Specific Professional skills (9)			
Interprofessional Perspectives/Respect (6)			Roles/Responsibilities (6)			
**OE5: ** ***What challenges or obstacles did you experience while working in the interprofessional student team?***						
No Challenges/Obstacles† (14)			No Challenges/Obstacles† (17)			
Patient Communication/Literacy (8)			Roles/Responsibilities† (12)			
Roles/Responsibilities (7)			Event Organization/Flow (7)			
Event Organization/Flow (6)			Patient Volume (-) (6)			
Interprofessional Communication (5)			Interprofessional Communication (5)			
**OE6: ** ***Did participating in this RAM clinic encourage you to pursue more rural healthcare experiences in the future? Please explain your answer***						
Yes† (62)			Yes† (59)			
Professional Satisfaction† (24)			Professional Satisfaction† (22)			
Patient/Community Need† (14)			Rural/Underserved Future Plans† (19)			
Impact (8)			Patient/Community Need† (14)			
From Rural/Similar Area (6)						
**OE7: ** ***Please include any further feedback, suggestions, or highlights***						
Student Volunteer Volume (5)			Event Organization/Flow† (18)			
			Professional Satisfaction (8)			

NOTES:

* *p* < 0.05 indicates a statistically significant difference between pre-event survey and post-event survey mean scores.

† Denotes major theme.

Themes are ordered top to bottom from most frequent to least frequent response. Number of responses for each question varied, as respondents were asked to choose and complete at least three open-ended questions, and a single respondent may have contributed multiple coded responses to a single question.

**Table 3:** Encounter and Intervention Summary

RAM Event Encounters	2017 Total	2018 Total	2019 Total	2020 Total
Total Unique Patients	830	779	778	239
Total Encounters	1102	1064	1092	276
Total Value of Care	$487,228	$556,768	$493,052	$132,872
Glasses	457	234	279	40
Extractions	1224	1209	1228	265
Fillings	243	307	246	78
Cleanings	84	104	61	29
Medical Exams	147	262	138	61
Naloxone Rescue Training	117	265	216	8
Medical Library Searches	210	118	22	15
Medical Library Deliverables (packets)	638	695	117	100
**Interprofessional Team Interventions**	**2017 Total**	**2018 Total**	**2019 Total**	**2020 Total**
*Skills Conducted*				
COVID-19 Screening & Temperature	–	–	–	404
Medication History	489	409	338	129
Blood Glucose Test Obtained	319	70	68	14
Blood Pressure Test Obtained	5	11	16	13
Health Screen Completed	225	210	353	117
Other Skills Conducted	123	128	79	53
*Education Provided*				
Blood Glucose Results Education	268*	30*	56*	4
Diabetes Education	157*	17	16*	3
Immunization Education	78*	27*	34*	25*
Diet/Exercise Education	78*	50*	15	4
Smoking Cessation Education	69*	36*	35*	21*
Anxiety/Depression Education	58	38*	28*	28*
Hypertension Education	27	13	5	4
Dental Health Education	12	5	11	43*
Discharge Medication Education	–	–	–	48*
Other Education Conducted	32	37	36	22
*Referrals Completed*				
Referral for Naloxone Rescue Training	36	5	32	8
Referral for On-site Immunization	177	22	153	98
Referral for On-site Hep C/HIV Screening	2	8	38	25
Referral for Medical Visit	0	3	33	28
Referral for Mental Health/Counseling	0	3	24	6
Referral for Women’s Health Services	0	1	38	26
Other Referrals Conducted	0	7	103	90
*Miscellaneous*	2	0	0	0
**Total of Logged Interventions**	**2,157**	**1,130**	**1,511**	**1,213**
**Interventions per Patient**	**2.6**	**1.5**	**1.9**	**5.1**

NOTES:

* Top five educational topics each year
