# Supplementary material for: Impact of Interprofessional Student Teams at a Remote Area Medical Event in Rural Appalachia
Source: J Appalach Health. 2023 Aug 1;5(2):66–84. doi: 10.13023/jah.0502.06 (PMC10629884; doi:10.13023/jah.0502.06)
Supplement: Supplementary file 1 [file 5.2.6_Flores_AdditionalFile.pdf]

Figure 1: Interprofessional Student Distribution

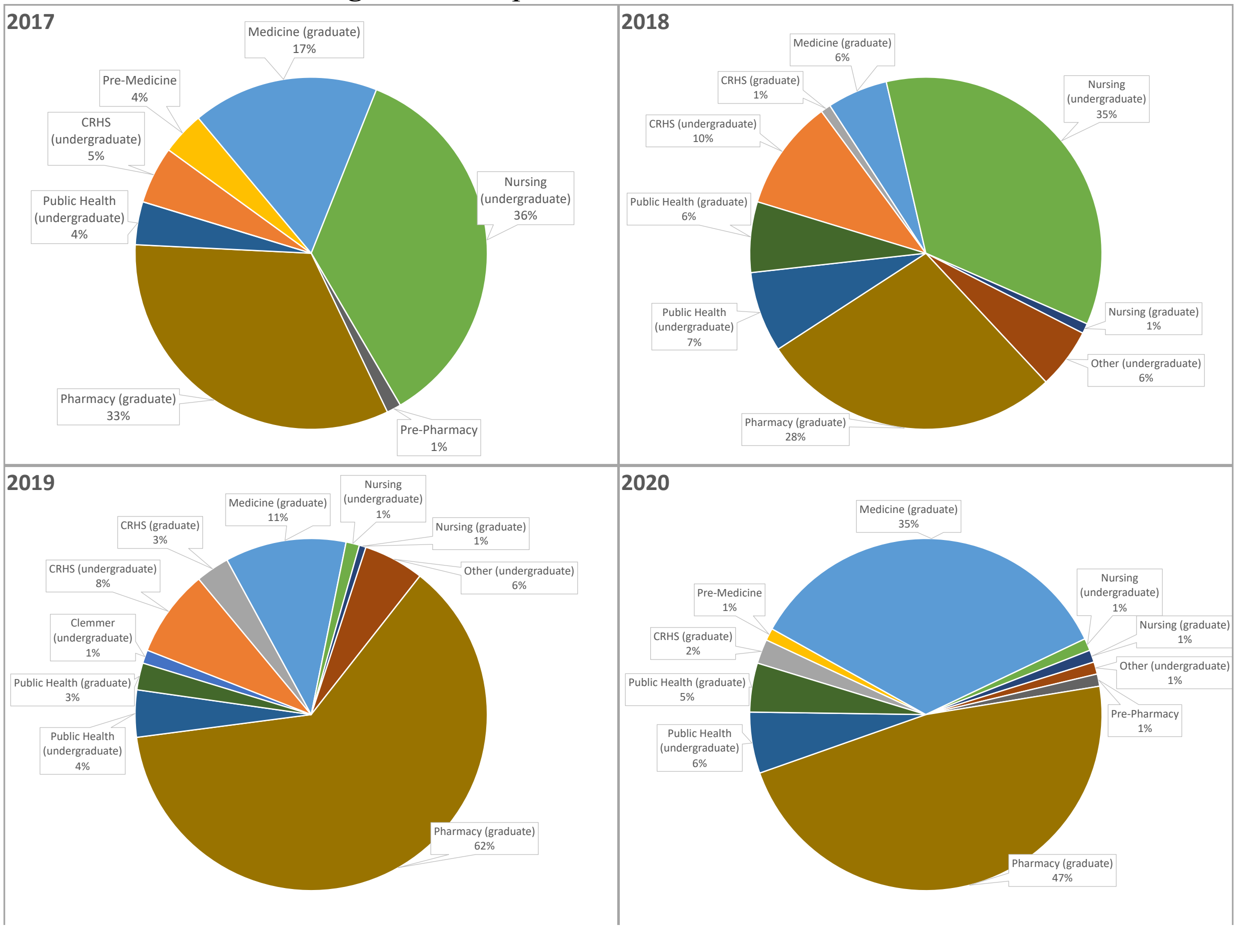

Figure 1 Caption: *2017 student volunteers N=76 (87 student experiences); 2018 student volunteers N=108 (124 student experiences); 2019 student volunteers N=162 (183 student experiences); 2020 student volunteers N=89 (93 student experiences); CRHS=College of Rehabilitative Health Sciences; Clemmer College=Counseling, Education, Leadership, and Sports degree programs*

Table 1. 2019 Student Demographics (N = 108) and 2020 Student Demographics (N=79)

		**2019**	**2020**
**Gender**	Male	33 (30.6%)	19 (24.1%)
	Female	75 (69.4%)	59 (74.7%)
	Other	-	1 (1.3%)
**Age group**	18 to 20	19 (17.6%)	4 (5.1%)
	21 to 25	67 (62%)	54 (68.4%)
	26 to 30	13 (12%)	11 (13.9%)
	31 to 35	6 (5.6%)	6 (7.6%)
	Over 35	3 (2.8%)	4 (5.1%)
**Hometown size**	Rural	29 (26.9%)	18 (22.8%)
	Small Town	49 (45.4%)	33 (41.8%)
	Suburb	21 (19.4%)	21 (26.6%)
	Large City	9 (8.3%)	7 (8.9%)
**Profession of study**	Allied Health	3 (2.8%)	1 (1.3%)
	Audiology	1 (0.9%)	0
	Medicine	12 (11.1%)	28 (35.4%)
	Nursing	(3.7%)	3 (3.8%)
	Pharmacy	67 (62%)	37 (46.8%)
	Physical Therapy	5 (4.6%)	0
	Public Health	7 (6.5%)	6 (7.6%)
	Speech Pathology	2 (1.9%)	1 (1.3%)
	Other	7 (6.5%)	3 (3.8%)
**Progress towards current degree**	Undergraduate first half of degree	8 (7.5%)	2 (2.5%)
	Undergraduate last half of degree	18 (16.8%)	7 (8.9%)
(2019: 107 responses 2020: 79 responses)	Graduate first half of degree	57 (53.3%)	39 (49.4%)
	Graduate last half of degree	24 (22.4%)	31 (39.2%)
**Immediate family member in a different health profession**	Yes	35 (32.4%)	24 (30.4%)
	No	73 (67.6%)	55 (69.6%)
**Number of past IPE experiences**	None	44 (40.7%)	17 (21.5%)
	1 to 3	53 (49.1%)	31 (39.2%)
	4 to 6	5 (4.6%)	12 (15.2%)
	7 to 9	0	8 (10.1%)
	10 or more	6 (5.6%)	11 (13.9%)
**Currently enrolled in ETSU IPE program**	Yes	71 (65.7%)	43 (54.4%)
	No	37 (34.3%)	36 (45.6%)
**Graduated from ETSU IPE program**	Yes	8 (7.4%)	25 (31.6%)
	No	100 (92.6%)	54 (68.4%)
**Past participation in RAM Interprofessional Teams**	Yes	16 (14.8%)	26 (32.9%)
	No	92 (85.2%)	53 (67.1%)

Table 1 Caption: *IPE = Interprofessional education*

Table 2. 2019 and 2020 Survey Results

**Quantitative Findings**						
	**2019 Pre-survey M (SD)**	**2019 Post-survey M (SD)**	**2019 *p*-value**	**2020 Pre-survey M (SD)**	**2020 Post-survey M (SD)**	**2020 *p*-value**
**SPICE-R2**	N=70			N=69		
Interprofessional Teamwork Factor	4.50 (0.72)	4.54 (0.72)	0.568	4.67 (0.43)	4.74 (0.41)	<0.001*
Roles and Responsibilities for Collaborative Practice Factor	4.00 (0.81)	4.09 (0.81)	0.374	4.13 (0.73)	4.49 (0.52)	<0.001*
Patient Outcomes from Collaborative Practice Factor	4.36 (0.76)	4.40 (0.77)	0.650	4.49 (0.55)	4.68 (0.50)	<0.001*
**ICCAS-R**	N=104			N=79		
Overall Composite Score	3.65 (0.80)	4.03 (0.73)	<0.001*	4.19 (0.65)	4.59 (0.50)	<0.05*
**Qualitative Themes**						
**November 2019**			**November 2020**			
**OE1:** ***What surprised you while volunteering at the Gray, Tennessee RAM event?***						
Patient/Community Need† (22)			Patient/Community Need† (17)			
Resources/Services† (13)			Resources/Services† (16)			
Volunteer Volume/Willingness† (12)			Patient Volume (-) (8)			
Impact (5)			Event Organization/Flow (7)			
Kindness (5)			Teams/Teamwork (5)			
Event Organization/Flow (5)			Volunteer Volume/Willingness (5)			
Patient Engagement (5)			COVID-19 Pandemic (5)			
Patient Variety/Diversity (5)						
**OE2:** ***What is something that you learned from participating in interprofessional student teams at the Gray, Tennessee RAM event?***						
Specific Professional Skills† (20)			Roles/Responsibilities† (15)			
Teams/Teamwork† (14)			Teams/Teamwork† (15)			
Patient/Community Need† (10)			Specific Professional Skills† (13)			
Roles/Responsibilities (6)			Impact (7)			
Volunteer Volume/Willingness (6)			Patient/Community Need (7)			
Interprofessional Communication (5)						
Resources/Services (5)						
**OE3:** ***How did working in an interprofessional student team change your idea of the roles of other health professionals?***						
Roles/Responsibilities† (22)			Roles/Responsibilities† (22)			
Interprofessional Perspectives/Respect† (17)			Interprofessional Perspectives/Respect† (15)			
Teams/Teamwork† (12)			Teams/Teamwork† (13)			
**OE4:** ***How did working in an interprofessional student team impact you as an individual within your chosen profession?***						
Professional Satisfaction† (20)			Professional Satisfaction† (15)			
Roles/Responsibilities† (13)			Interprofessional Perspectives/Respect† (11)			
Teams/Teamwork (7)			Teams/Teamwork† (10)			
Interprofessional Communication (6)			Specific Professional skills (9)			
Interprofessional Perspectives/Respect (6)			Roles/Responsibilities (6)			

**OE5:** ***What challenges or obstacles did you experience while working in the interprofessional student team?***	
No Challenges/Obstacles† (14)	No Challenges/Obstacles† (17)
Patient Communication/Literacy (8)	Roles/Responsibilities† (12)
Roles/Responsibilities (7)	Event Organization/Flow (7)
Event Organization/Flow (6)	Patient Volume (-) (6)
Interprofessional Communication (5)	Interprofessional Communication (5)
**OE6:** ***Did participating in this RAM clinic encourage you to pursue more rural healthcare experiences in the future? Please explain your answer***	
Yes† (62)	Yes† (59)
Professional Satisfaction† (24)	Professional Satisfaction† (22)
Patient/Community Need† (14)	Rural/Underserved Future Plans† (19)
Impact (8)	Patient/Community Need† (14)
From Rural/Similar Area (6)	
**OE7:** ***Please include any further feedback, suggestions, or highlights***	
Student Volunteer Volume (5)	Event Organization/Flow† (18)
	Professional Satisfaction (8)

Table 2 Caption: **$p < 0.05$ indicates statistically significant difference between pre-survey and post-survey mean scores. Themes are ordered top to bottom from most frequent to least frequent response; †Denotes major theme; Number of responses for each question varied as respondents were asked to choose and complete at least 3 open-ended questions and a single respondent may have contributed multiple coded responses to a single question.*

Table 3. Encounter and Intervention Summary

**RAM Event Encounters**	**2017 Total**	**2018 Total**	**2019 Total**	**2020 Total**
Total Unique Patients	830	779	778	239
Total Encounters	1102	1064	1092	276
Total Value of Care	$487,228	$556,768	$493,052	$132,872
Glasses	457	234	279	40
Extractions	1224	1209	1228	265
Fillings	243	307	246	78
Cleanings	84	104	61	29
Medical Exams	147	262	138	61
Naloxone Rescue Training	117	265	216	8
Medical Library Searches	210	118	22	15
Medical Library Deliverables	638 handouts	695 handouts	117 packets	100 packets
**Interprofessional Team Interventions**	**2017 Total**	**2018 Total**	**2019 Total**	**2020 Total**
Skills Conducted				
COVID-19 Screening & Temperature	-	-	-	404
Medication History	489	409	338	129
Blood Glucose Test Obtained	319	70	68	14
Blood Pressure Test Obtained	5	11	16	13
Health Screen Completed	225	210	353	117
Other Skills Conducted	123	128	79	53
Education Provided				
Blood Glucose Results Education	268*	30*	56*	4
Diabetes Education	157*	17	16*	3
Immunization Education	78*	27*	34*	25*
Diet/Exercise Education	78*	50*	15	4
Smoking Cessation Education	69*	36*	35*	21*
Anxiety/Depression Education	58	38*	28*	28*
Hypertension Education	27	13	5	4
Dental Health Education	12	5	11	43*
Discharge Medication Education	-	-	-	48*
Other Education Conducted	32	37	36	22
Referrals Completed				
Referral for Naloxone Rescue Training	36	5	32	8
Referral for On-site Immunization	177	22	153	98
Referral for On-site Hep C/HIV Screening	2	8	38	25
Referral for Medical Visit	0	3	33	28
Referral for Mental Health/Counseling	0	3	24	6
Referral for Women’s Health Services	0	1	38	26
Other Referrals Conducted	0	7	103	90
Miscellaneous	2	0	0	0
**Total of Logged Interventions**	**2,157**	**1,130**	**1,511**	**1,213**
**Interventions per Patient**	**2.6**	**1.5**	**1.9**	**5.1**

Table 3 Caption: **Top 5 educational topics each year*
